# Hesperidin suppresses ERS-induced inflammation in the pathogenesis of non-alcoholic fatty liver disease

**DOI:** 10.18632/aging.203817

**Published:** 2022-02-10

**Authors:** Qi Xie, Shuqing Gao, Min Lei, Zengning Li

**Affiliations:** 1 Department of Nutrition, The Fourth Hospital of Hebei Medical University, Shijiazhuang 050011, China; 2 Department of Nutrition, The Third Hospital of Hebei Medical University, Shijiazhuang 050000, China; 3 Hebei Province Key Laboratory of Nutrition and Health, The First Hospital of Hebei Medical University, Shijiazhuang 050000, China; 4 Department of Nutrition, The First Hospital of Hebei Medical University, Shijiazhuang 050000, China

**Keywords:** hesperidin, non-alcoholic fatty liver disease, endoplasmic reticulum stress, inflammation

## Abstract

Objective: The current study aimed to establish a non-alcoholic fatty liver disease (NAFLD) model using HFD-fed SD rats and FFA-stimulated human THP-1 cells to examine whether hesperidin (HSP) plays a role in endoplasmic reticulum stress (ERS)-induced inflammation in the pathogenesis of NAFLD.

Methods: Oil red O staining was used to determine the effect of HSP on hepatic steatosis in rat liver tissues. Differentially expressed genes (DEGs) were subjected to functional enrichment analysis by bioinformatics. Western blotting was used to detect the protein expression of GRP94, ATF6, ATF4, p-PERK, p-IRE1α, IL-1β, IL-6, and TNF-α in liver tissues and THP-1 cell lines, and the expression of GRP94 and p-PERK *in vitro* was detected through immunofluorescence staining.

Results: HSP significantly decreased the weight gain, hepatic steatosis but not serum lipid profile and suppressed the serum levels of inflammatory factors in HFD-fed rats. It was revealed by bioinformatics analysis that the inflammatory response and IRE1α activation were enriched signaling pathways in NAFLD. The expression of ERS-related biomarkers, GRP94, ATF6, ATF4, p-PERK and p- IRE1α, was significantly suppressed by HSP *in vivo* and *in vitro*. Moreover, the inflammatory markers, including IL-1β, IL-6, and TNF-α, were also decreased by HSP *in vivo* and *in vitro*. Immunofluorescence staining exposed that the expression of GRP94 and p-PERK was decreased by HSP in vitro.

Conclusion: HSP may suppress ERS-induced inflammation in the pathogenesis of NAFLD.

## INTRODUCTION

Non-alcoholic fatty liver disease (NAFLD) is a common chronic hepatic disease with a series of liver disorders, eventually resulting in cirrhosis and hepatocellular carcinoma [1, 2]. Owing to the global obesity pandemic, the prevalence of NAFLD is continually growing worldwide, and a fast rise in NAFLD prevalence has also been perceived in China [3, 4]. Notably, due to the increasing growth in childhood obesity and more weakness to genomic and ecological reasons, NAFLD currently influences more and more pediatric populations [5, 6]. Consequently, NAFLD is swiftly becoming a leading public health problem worldwide [7]. Nevertheless, no applicable drug therapeutic strategy for NAFLD has been recognized. Therefore, it is crucial to investigate the molecular pathophysiology of NAFLD to explore the novel treatment of NAFLD.

Recent studies have confirmed that endoplasmic reticulum stress (ERS) and inflammation play critical roles in the development of NAFLD [8–10]. ER is an essential cellular organelle that regulates protein synthesis, folding, and post-translational modification [11–13]. ER is exquisitely sensitive to diverse stimuli and exhibits aggregation of unfolded or misfolded proteins in the lumen, resulting in perturbations in ER homeostasis [14–16]. If the aggregation of misfolded or unfolded protein load surpasses the folding capability of ER, unfolded protein response (UPR) will be activated. The ER-membrane receptors mediate the three pathways in the UPR process: pancreatic ER kinase (PKR)-like ER kinase (PERK), activating transcription factor-6 (ATF6), and inositol requiring protein 1 (IRE1), all of which are dominated by78 kDa glucose-regulated protein (GRP78) [17–19]. The activation of IRE-1α and PERK pathways contributes to inflammatory mediators [20, 21]. Under ERS, PERK phosphorylates the α subunit of the eukaryotic translation initiation factor-2 (eIF2α), thereby activating the nuclear transcription factor of the NF-κB pathway [22]. Then activated NF-κB amplifies the production of inflammatory factors, tumor necrosis factor-α (TNF-α), and interleukin-6 (IL-6) [23, 24].

Hesperidin (5, 7, 3′-trihydroxy-4′-methoxy-flavanone7-rhamno glucoside, HSP) is a flavonoid glycoside extensively found in the fruits of the genus Citrus and vegetables, which is known for its valuable antioxidant activities, anti-inflammatory, anti-cancer, and other advantageous healthiness effects [25, 26]. In recent years, numerous studies have suggested that HSP exerts regulatory effects in many diseases. Silvia et al. indicated that HSP can induce non-apoptotic cell death and acts as a promising chemotherapeutic agent for liver cancer [27]. Meanwhile, HSP was found to be a therapeutic target for diabetic patients [28]. Likewise, it was observed that HSP functions as a tumor suppressor in breast cancer, suggesting its potential therapeutic value [29]. Ratana et al. indicated that HSP induces apoptosis of human hepatocellular carcinoma HepG2 cells via intrinsic and extrinsic pathways [30]. Yusuf et al. indicated that HSP may be used as a meaningful prophylactic agent and a promising adjuvant treatment option for SARS-CoV-2 infection [31]. Simultaneously, HSP is a valuable factor for liver injury protection and a potential treatment for liver I/R injury [32]. However, whether HSP is indeed involved in NAFLD has not yet been investigated.

Consequently, the objective of the current study is to assess the effect of HSP on NAFLD. In addition, the ERS signaling pathway and inflammatory response were estimated to investigate the molecular mechanism of HSP on NAFLD.

## METHODS

### Animals and model of NAFLD

Male 6-8-week-old SD rats, weight 150 ± 20 g, were fed in the laboratory animal center of the fourth affiliated hospital of Hebei medical university. All animals were adaptively fed for one week, and kept in an SPF laboratory (temperature 25 ± 2°C, humidity 5% ± 4%, 12 h: 12 h light/dark cycle). The experimental animals had free access to water and food for one week. Rats were fed with normal diet (ND) and high-fat diet (HFD) (containing 1.2% cholesterol, 15% lard, 20% sucrose, 0.2% sodium cholate, 0.6% dicalcium phosphate, 0.4% limestone, 10% casein, and 0.4% premix mixed with the normal diet), and gavaged with the same volume of normal saline and HSP (200 mg/kg.bw) for 12 weeks to induce NAFLD. The rats were fasted for 12 h before changing the feed. At the end of the experiment, blood samples were taken from the anesthetized rats and liver tissues were dissected from rats sacrificed by cervical dislocation.

### Oil red-O staining

Frozen sections of rat liver were made (−15°C). After fixation for 15 minutes, the slides were immersed in the Oil Red O stain for 15 minutes, rinsed with isopropanol (at a concentration of 60%), re-stained with Mayer hematoxylin for 2 minutes, and washed with running water at last. The lipid content = the Oil Red O stained area/lumen area.

### Determination of serum lipids

The indexes of serum lipid metabolism were detected, including total cholesterol, triglyceride (TC), and low-density lipoprotein (LDL) in serum according to the operating instructions of the kit using an automatic biochemical analyzer.

### ELISA of TNF a, IL-6, IL-1β and fatty acids (FFAs)

After centrifugation (at 3000 r·min-1 for 10 min) of the blood at 4°C, the serum was collected. An ELISA kit was used to detect tumor necrosis factor-alpha (TNF-α), interleukin (IL)-6, IL-1β and FFA according to the instructions.

### THP-1 macrophage culture

THP-1 cells (human monocytes) were cultured in an RPMI-1640 cell culture medium until the logarithmic growth phase. Then the cells were inoculated into the complete medium (cell concentration was adjusted to 5105), added with 2 mL of PMA (100 ng/ mL) and cultured for 24 h. The cell status was observed and photographed. The observation that single round, suspended cells gradually formed into fusiform or irregular shapes and adhered cells with pseudopod formation indicated that THP-1 cells had differentiated into macrophages.

### The displaced fatty acids (FFAs) stimulate macrophages

The differentiated macrophages were cultured in a DMEM with 10% fetal bovine serum (FBS) for 12 h. Then the culture medium was replaced with a high-glucose medium with 5% FBS. 24 h later, the cells were washed with PBS, cultured with new high-glucose medium with 5% FBS, and added with FFA to make the final concentration of FFA of 0.5 mmol/L, HSP of 140 mg/L, and 1 μM of thapsigargin, followed by culture for 48 h.

### THP-1 and HepG2 co-culture

Extraction of macrophage conditioned medium: Human monocyte cell line (THP-1) and hepatic cell line (HepG2) were cultured in 1640 medium containing 10% FBS and 1% double antibody. The cells in logarithmic phase were added, and co-cultured THP-1 and HepG2 cells were washed by PBS, cultured with non-FBS serum with or without 0.5 mmol/L FFA, 140 mg/L HSP and 1 μM thapsigargin for another 24 hours and the proteins were collected and tested by Western blotting.

### Western blotting analysis

Western blotting was used to detect protein expression levels. After the liver tissue was ground, the lysate was melted at room temperature to extract the proteins in the liver tissue. The proteins were determined by the BCA method. After the determination, the proteins were separated by 8–10% SDS-PAGE and then transferred to the PVDF membrane. After blocking with 5% skim milk powder for 2 h, the proteins were incubated with the primary antibody overnight, and with the secondary antibody for 1 h, followed by exposure. Quantitative analysis of the Western blot was carried out using the ImageJ software (Version 1.52 s for Windows).

### Immunofluorescence double staining

After 2 × 10^3^ THP-1 single-cell suspension was added into a static 24-pore plate and treated with or without FFAs, HSP and thapsigargin, the prepared slides were placed on the droplets overnight. The dishes and slides were glued together. After 10 minutes, the liquid was absorbed and dried in the ultra-clean table. After the cells were washed with PBS, they were fixed with fixative for 10 minutes, incubated with hydrogen peroxide for 30 minutes, and sealed using goat serum for 30 minutes. After the liquid was discarded, the cells were directly incubated with the antibodies (mouse polyclonal to GRP78, Cat No. 60012-2-Ig and rabbit anti-PERK (phospho T982), ab192591) for 8 hours at 4°C away from light and washed, and then incubated for 45 minutes with Donkey anti-rabbit IgG H&L (FITC) for PEKR (phospho T982) staining and goat anti-mouse IgG H&L (PE/Cy5.5^®^)-labeled secondary antibodies for GRP78 staining then washed away the second antibodies avoid light exposure, followed by DAPI re-staining, cleaning and sealing. Finally, the images were observed and collected under a fluorescence microscope.

### Statistical analysis

SPSS 19.0 and GraphPad Prism 6 software were used for statistical analysis. The measurement data were analyzed by ANOVA or *t*-test, the count data and categorical data were analyzed by chi-square test or rank-sum test, and the correlation analysis was performed by grade correlation analysis, with *P* < 0.05 as a significant difference.

### Ethics statement

All animal experiments were approved by The Fourth Affiliated Hospital of Hebei Medical University.

## RESULTS

### HSP suppressed HFD-induced body weight gain and hepatic steatosis but not serum lipid profile in the rat

**Figure 1:**
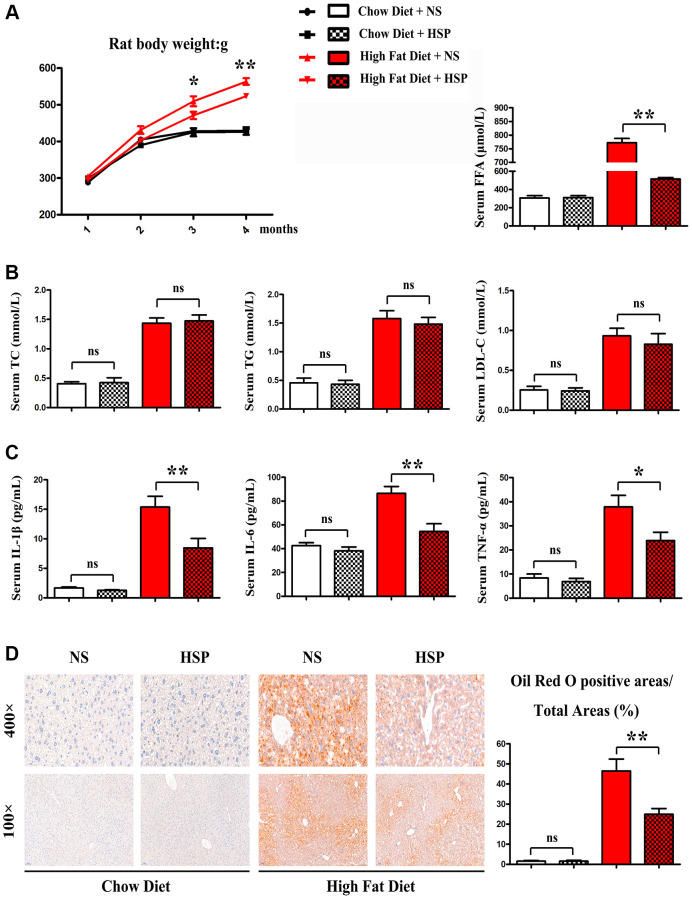
**HSP suppressed HFD-induced body weight gain and hepatic steatosis but not serum lipid profile.** SD rats were fed with CD or HFD for 4 months and were randomly divided into CD + NS group, CD + HSP group, HFD + NS group, and HFD + HSP group. (**A**) The body weight of the rat was evaluated. Data were presented as mean ± SEM. ^**^P < 0.05. (**B**) The levels of serum TC, TG, and LDL, FFA were examined (**C**) the levels of serum IL-1β, IL-6, TNF-α. Data were presented as mean ± SEM. (**D**) Histological analysis of hepatic steatosis stained with Oil Red O staining.

We employed the SD rat to investigate the roles of HSP in the NAFLD. SD rats were fed with CD or HFD for 4 months and randomly divided into CD + NS group, CD+HSP group, HFD+NS group, and HFD + HSP group (*N* = 7/group). As shown in Figure 1A, the HFD-fed rat noticeably gained body weight compared to the CD-fed rat.

Furthermore, HFD markedly enhanced the levels of serum TC TG and LDL. However, no significant differences were observed in serum TC, TG, and LDL between CD + NS group and CD+HSP group and between HFD + NS group and HFD + HSP group (Figure 1B). Next the effects of HSP on hepatic steatosis in the hepatic sections of SD rats were examined in the four groups. Oil Red O staining indicated a notable rise of lipid deposition in the hepatic sections of HFD-fed rats compared with CD-fed rats (Figure 1C). More importantly, HSP significantly decreased the increase in lipid deposition in liver tissues (Figure 1C). Oil red O positive areas/total areas for lipid deposition revealed that HFD markedly increased the lipid deposition in livers of rats compared with that in CD-fed rats, and such an increase was significantly diminished by HSP (*P* < 0.05, Figure 1D). These findings revealed that HSP suppressed HFD-induced body weight gain and hepatic steatosis but not serum lipid profile in rats.

### Identification of DEGs in two NAFLD-related GEO datasets

**Figure 2:**
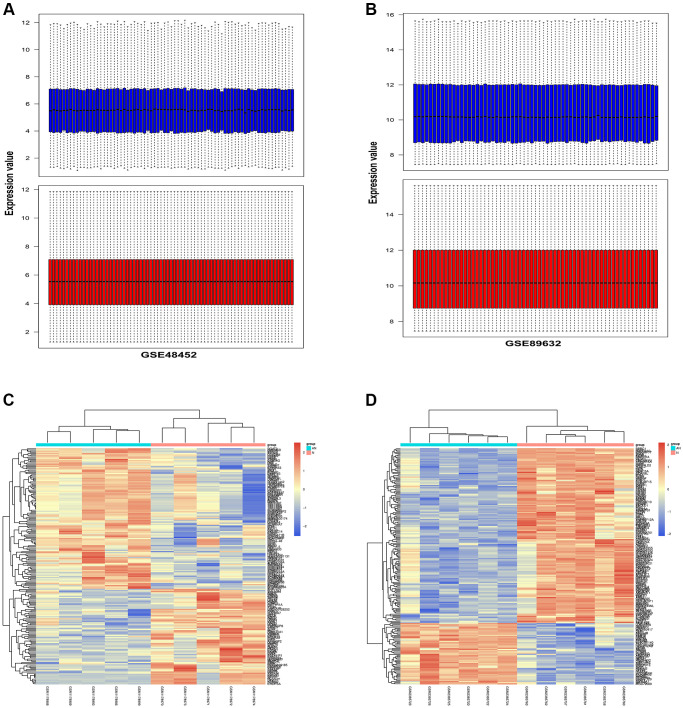
**Identification of DEGs in two NAFLD-related GEO datasets.** NAFLD-related gene expression profiles GSE48452 and GSE89632 were employed to detect the DEGs between NAFLD patients and healthy donors. (**A**) The results of pre-standardization and post-standardization based on GSE48452 were exhibited. (**B**) The results of pre-standardization and post-standardization based on GSE89632 were exhibited. (**C**) Heap map exhibited the upregulated and downregulated DEGs in GSE48452. X-axis exhibited samples. Y-axis exhibited the genes. The left dendrogram exhibited gene clustering. The upper dendrogram exhibited the sample clustering. (**D**) Heap map exhibited the upregulated and downregulated DEGs in GSE89632. X-axis exhibited samples. Y-axis exhibited the genes. The left dendrogram exhibited gene clustering. The upper dendrogram exhibited the sample clustering.

To explore the biological process in NAFLD, comparative proteomic bioinformatics analysis was performed in this study. NAFLD-related gene expression profiles GSE48452 and GSE89632 were employed to detect the DEGs between NAFLD patients and healthy donors. The gene expression profiles of the GSE48452 and GSE89632 datasets were harmonized using quartile division, and the results of pre-standardization and post-standardization were exhibited in Figure 2A, 2B. Then, we applied the R software to obtain DEGs according to the data from GSE48452. The DEG distribution in GSE48452 was exhibited in a heat map (Figure 2C), including 247 upregulated ones and 165 downregulated ones. Similarly, another heat map was screened out with 307 upregulated DEGs and 298 downregulated DEGs based on GSE89632 (Figure 2D).

### Functional annotation and GSEA analysis of DEGs

**Figure 3:**
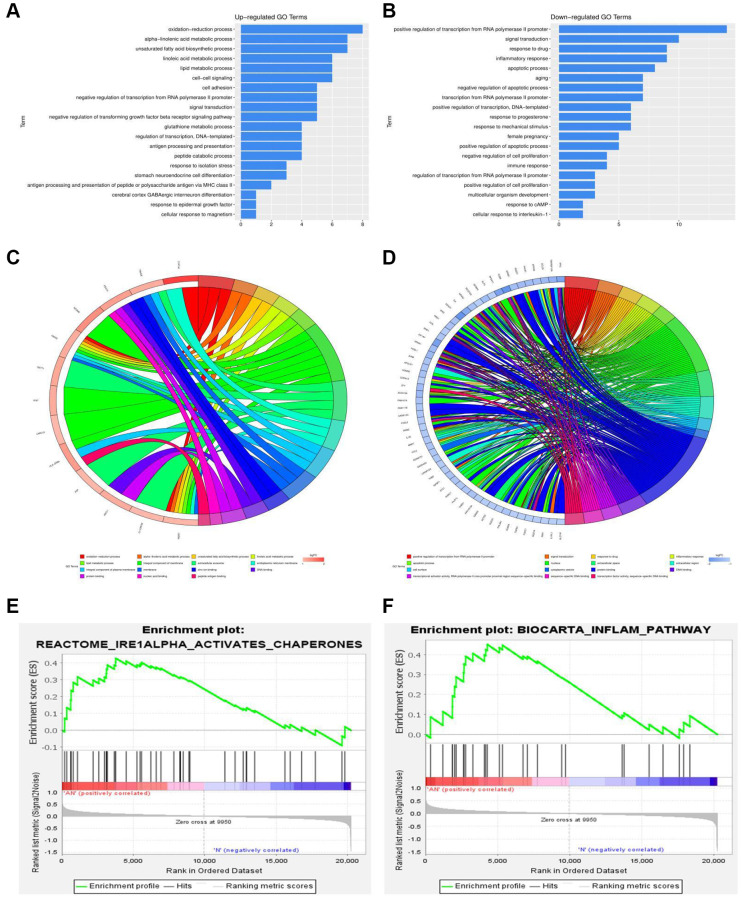
**Functional annotation and GSEA analysis of DEGs.** (**A**, **B**) GO and KEGG enrichment analysis of DEGs associated with NAFLD-related GSE48452. (**C**, **D**) GO and KEGG enrichment analysis of DEGs associated with NAFLD-related GSE89632. (**E**, **F**) GSEA of DEGs associated with NAFLD-related GSE48452 and GSE89632.

Gene set enrichment analysis (GSEA) was performed to assess the gene expression profiles (GSE48452 and GSE89632) as biological relevance. GO enrichment analysis was performed to explore the biological functions and pathways of all the DEGs. According to the data from GSE48452, the GO and KEGG enrichment pathway analysis for biological processes were mainly involved in the oxidation-reduction process, positive regulation of IRE1-mediated unfolded protein response, ATF1-ATF4 transcription factor complex, inflammatory response, apoptotic process, and immune response signaling pathways (Figure 3A, 3B). Results showed that most DEGs from GSE48452 and GSE89632 were enriched in the inflammatory response and IRE1α activation signaling pathway (Figure 3E, 3F). Moreover, most pathways in the GO and KEGG enrichment analysis for GSE89632 were associated with the oxidation-reduction process, lipid metabolic process, extracellular exosome, inflammatory response, apoptotic process, extracellular exosome, and ER membrane signaling pathways (Figure 3C, 3D). Collectively, it is hypothesized by these findings that the effects of HSP on NAFLD may be realized via the ERS-induced inflammatory response signaling pathway.

### HSP attenuated ERS-induced inflammation in rat liver

**Figure 4:**
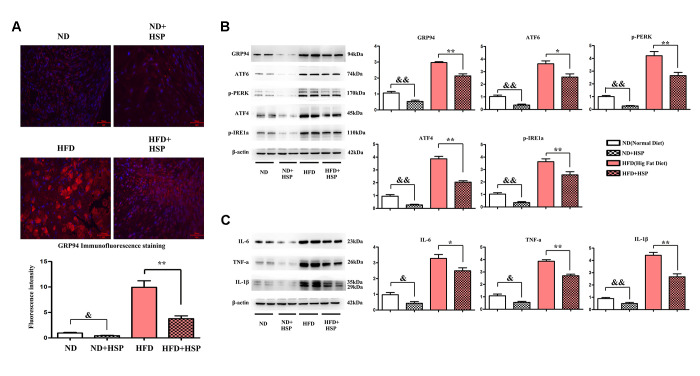
**HSP could attenuate ERS-induced inflammation in the liver.** (**A**) Immunofluorescence staining was performed to examine the expression of GRP94 in liver tissues. (**B**) Western blotting analysis was performed to examine the expression of GRP94, ATF6, ATF4, p-PERK, and p-IRE1α in liver tissues. Data were presented as mean ± SEM. ^**^P < 0.05, HFD + HSP vs. HFD; ^&&^*P* < 0.05, ND + HSP vs. ND. (**C**) Western blotting analysis was performed to examine the expression of IL-1β, IL-6, and TNF-α. Data were presented as mean ± SEM. ^**^P < 0.05, HFD + HSP vs. HFD; ^&&^*P* < 0.05, ND + HSP vs. ND.

We next examined the effects of HSP on the ERS in liver tissues of the rat. Immunofluorescence staining was performed to investigate the expression of GRP94 in liver tissues. As shown in Figure 4A, the expression of GRP94 was significantly increased in the liver tissues of HFD-fed rats compared with ND-fed rats (*P* < 0.05). Moreover, the GRP94 positive area was significantly decreased after HSP treatment. The fluorescence intensity of GRP94 was increased by HFD, whereas HSP could decrease the expression of GRP94 (*P* < 0.05, Figure 4A). The abundance of ERS-related proteins was evaluated by Western blotting. The protein expression levels of GRP94, ATF6, ATF4, p-PERK, and p-IRE1α in liver tissues of the HFD group were higher than those in the ND group (Figure 4B). The relative protein expression of these molecules was significantly increased in the liver tissues of ND+HSP-treated rats compared with that in ND-fed rats (Figure 4B, *P* < 0.05). Similarly, the relative protein expression of these molecules was significantly increased in HFD + HSP-treated rats compared with that in HFD-fed rats (Figure 4B, *P* < 0.05), suggesting that HSP can attenuate ERS in the liver, which was further verified by the marked increase in protein expression of GRP94, ATF6, ATF4, p-PERK, and p-IRE1α in liver tissues. Cytokines implicated in inflammatory responses including IL-1β, IL-6, and TNF-α were all increased in the liver tissues in HFD group compared with those in ND group, whereas they were decreased in HFD + HSP group compared with those in HFD group (Figure 4C). The relative protein expression of FFA, IL-1β, IL-6, and TNF-α was significantly decreased in ND + HSP-treated rat compared with that in ND-fed rats (Figure 4C, *P* < 0.05). Furthermore, the expression of IL-1β, IL-6, and TNF-α was also decreased in HFD + HSP-treated rat compared with that in HFD-fed rats (Figure 4C, *P* < 0.05). The results suggested that HSP can attenuate ERS-induced inflammation in the liver.

### HSP attenuated ERS in human THP-1 cells

**Figure 5:**
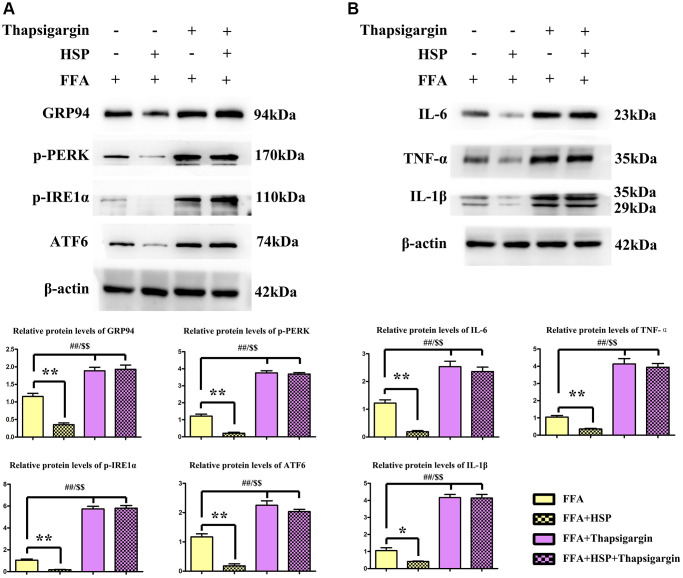
**HSP attenuates ERS in human THP-1 cells.** As described in the method, FFA-stimulated human THP-1 cells were employed to establish the NAFLD model. Thapsigargin is a well-described ERS inducer successfully used in various cell lines. (**A**) Western blotting analysis was performed to examine the expression of GRP94, ATF6, ATF4, p-PERK, and p-IRE1α in human THP-1 cells. (**B**) Western blotting analysis was performed to examine the expression of IL-1β, IL-6, and TNF-α.

Macrophages have been shown to play crucial roles in inflammation, which promotes hepatic steatosis in NAFLD [33]. In the current study, FFA-stimulated human THP-1 cells were employed to establish the NAFLD model as described above. Thapsigargin is a well-described ERS inducer that was successfully used in various cell lines. After pretreatment with HSP for 24 hours, macrophages were induced with FFA for another 24 hours, and the protein expression levels of GRP94, ATF6, ATF4, p-PERK, p-IRE1α were examined by Western blotting (Figure 5A). The results revealed that HSP decreased GRP94, ATF6, ATF4, p-PERK, and p-IRE1α expression in FFA-stimulated THP-1 cells (Figure 5A, *P* < 0.05). Thapsigargin significantly enhanced the expression of GRP94, ATF6, ATF4, p-PERK, and p-IRE1α. Cytokines implicated in inflammatory responses including IL-1β, IL-6, and TNF-α were all decreased by HSP in FFA-stimulated THP-1 cells (Figure 5B, *P* < 0.05). Thapsigargin also enhanced the expression of these inflammatory cytokines. The results suggested that HSP can attenuate ERS-induced inflammation in FFA-stimulated THP-1 macrophages.

### HSP attenuated ERS in human THP-1 cells

**Figure 6:**
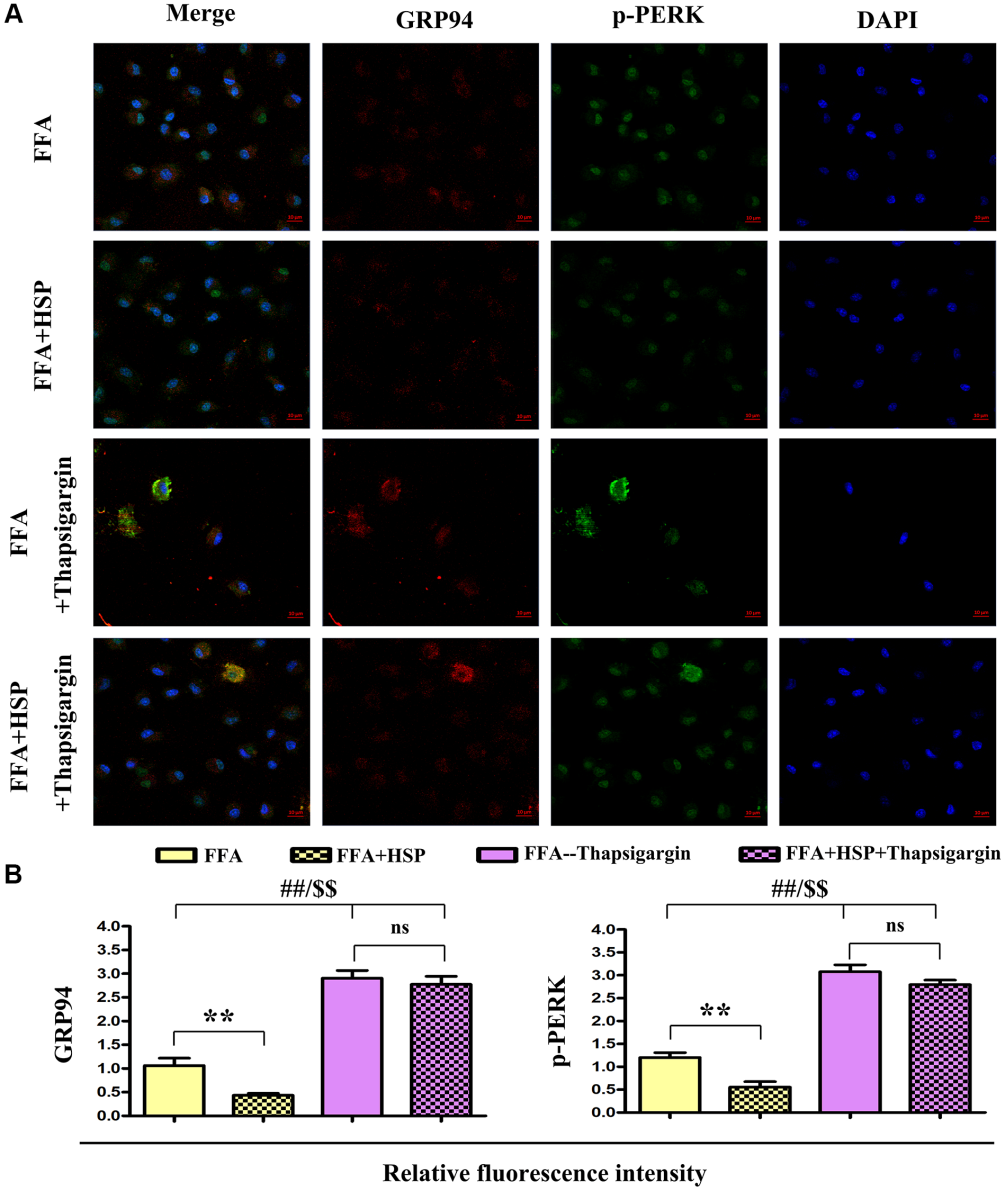
**HSP attenuates ERS in human THP-1 cells.** As described in the method, FFA-stimulated human THP-1 cells were employed to establish the NAFLD model. Thapsigargin was used to simulate the *in vitro* ERS model. (**A**) Immunofluorescence staining was performed to examine the expression of GRP94 and p-PERK with or without HSP. (**B**) Relative fluorescence intensity of GRP94 and p-PERK. Data were presented as mean ± SEM. ^**^P < 0.05, FFA + HSP vs. FFA.

GRP94 and p-PERK were examined by double immunofluorescence staining. As shown in Figure 6A, the levels of GRP94 and p-PERK were decreased in the HSP+FFA group compared with those in FFA group. Furthermore, thapsigargin could significantly increase the expression of GRP94 and p-PERK. Relative fluorescence intensity showed that HSP significantly downregulated the expression of GRP94 and p-PERK in FFA-stimulated macrophages (Figure 6A, *P* < 0.05), but thapsigargin did not induce any apparent change between HSP+FFA and FFA groups, and the statistical chart were shown in Figure 6B, *P* < 0.05. The results suggested that HSP can attenuate ERS in the liver in FFA-stimulated human THP-1 cells.

### HSP decreased the FFA induced hepatic lipid synthesis proteins in THP-1 and HepG2 co-cultured environment

**Figure 7:**
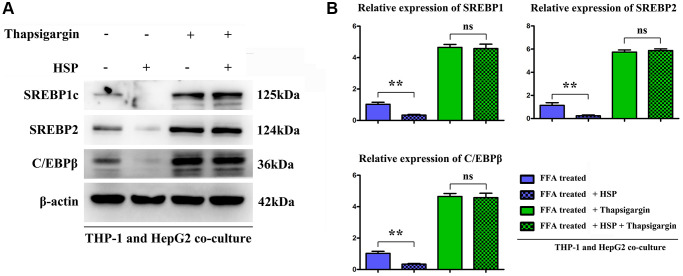
**HSP suppressed macrophages modulated the lipid metabolism related proteins expression in hepatocytes cells.** FFA-stimulated THP-1 cells treated with HSP or Thapsigargin, and the associated cell supernatant were used to stimulate hepatocytes. The lipid metabolism associated proteins: SREBP-1/-2 and C/EBP β were tested by Western blotting, as shown in (**A**) and (**B**). Data were presented as mean ± SEM. ^**^P < 0.05 control vs. HSP.

To identified the effect of HSP on hepatic lipid synthesis, the hepatic lipogenesis modulatory proteins CCAAT/enhancer-binding protein (C/EBP), and sterol regulatory element-binding protein 1c (SREBP-1c) and 2 were tested. FFA is the major causative factor for the progression of NAFLD. The results showed the HSP inhibited the expression of lipogenic protein compared with control group, and the ERS inducer thapsigargin significantly increased the lipogenic protein and there was no significant difference in thapsigargin between control and HSP groups (Figure 7), indicating the HSP suppresses the hepatic lipid accumulation by inhibition on ERS.

## DISCUSSION

NAFLD is a liver disease tightly correlated to ERS and inflammatory response, which may develop from hepatic steatosis to steatohepatitis, liver cancer, and even cardiovascular diseases [34]. NAFLD is linked to the development of left ventricular dysfunction, atherosclerotic CV disease, and ischemic stroke, according to Claudio et al. [35]. In China, the incidence of NAFLD has been reported to be on the rise, with a growing trend towards the pediatric population. According to Ashok et al., increasing childhood obesity is linked to an increased risk of childhood NAFLD [36]. As a result, it’s critical to figure out how NAFLD works and what may be done to prevent it.

Hesperidin (HSP), a potent flavonoid largely found in citrus fruits, is a novel potential therapeutic application for many diseases [37]. According to recent research, HSP’s protective effects are linked to antioxidant and anti-inflammatory properties [38]. According to a recent study, HSP may protect the liver from inflammation and oxidative stress-induced natural and manmade toxins [39]. HSP may protect against liver I/R injury by activating the Akt pathway, which reduces liver oxidative stress, suppresses inflammation, and prevents hepatocyte death, according to another study [9]. HSP can protect rat livers against nZnO-induced oxidative damage, according to Sabah et al. [40]. In a previous work, HDN was found to protect rat liver and kidney against CCl4-induced oxidative damage [1]. However, as far as we know, the influence of HSP in the NAFLD, as well as the relationship between HSP and ERS-induced inflammation in NAFLD, has not been previously described. Here, we aimed to explore the effects of HSP on the ERS and inflammation *in vivo* and *in vitro*.

The HSP doses in this study were 2000 mg/kg. Two types of diets (CD and HFD) were employed for the rat. The NAFLD rat models with HFD were successfully established in the present study. Oil Red O staining indicated a notable rise of lipid deposition in the hepatic sections of HFD-fed rats compared with CD-fed rats. Additionally, HSP administration significantly weaken the increase in lipid deposition in livers of HFD-fed rats. Oil red O positive areas/total areas for lipid deposition further revealed that HFD markedly increased the lipid deposition in livers of rats compared with that in CD-fed rats, and HSP significantly diminished this increase in livers of rat, implying that HSP suppresses HFD-induced hepatic steatosis. The analysis of body weight showed that the HFD-fed rat noticeably gained body weight compared with the CD-fed rat. Moreover, HFD-fed rats exhibited markedly higher levels of serum TC, TG, and LDL than CD-fed rats. However, no significant differences were observed in the levels of serum TC, TG, and LDL between the CD + NS group and CD + HSP group and between the HFD + NS group and HFD + HSP group. Based on these findings, it is concluded that HSP suppresses HFD-induced body weight gain and hepatic steatosis but not serum lipid profile.

A comparative proteomic bioinformatics analysis was used in this study to look at the biological processes that contribute to the development of NAFLD. From the GEO database, two NAFLD-related gene expression profiles (GSE48452 and GSE89632) were chosen and downloaded. With the use of R software, the DEGs between NAFLD patients and healthy donors were discovered. Using quartile division, the gene expression profiles of the two datasets were harmonized, and the pre- and post-standardization results were displayed. To display the DEGs in GSE48452 and GSE89632, heatmaps with clustering analysis were screened out. In addition, we used GO and KEGG enrichment analysis to investigate all of the DEGs’ biological activities and pathways. The majority of GSE48452 DEGs were discovered to be involved in the positive regulation of the IRE1-mediated unfolded protein response, the ATF1-ATF4 transcription factor complex, and the inflammatory response signaling pathways. GO and KEGG pathway enrichment analysis for GSE89632 was associated with the oxidation-reduction process and ER membrane signaling pathways. Gene set enrichment analysis (GSEA) was performed to assess the gene expression profiles as biological relevance. Results showed that most DEGs from the two datasets were enriched in the inflammatory response and IRE1α activation signaling pathway. Based on the bioinformatics analysis findings, it is hypothesized that the effects of HSP on NAFLD may be realized via the ERS-induced inflammatory response signaling pathway.

ER is a multifunctional organelle that regulates many cellular processes and plays a critical role in developing NAFLD [41]. ER dysfunction exhibited the overload of unfolded or misfolded proteins in the cell, resulting in ERS. In the initial step of ERS, the expression of ER molecular chaperone protein and 78 kDa glucose-regulated protein (GRP78) are upregulated, leading to the activation of PERK, ATF6, and IRE1α [42]. Our study found that the protein expression of GRP94, ATF6, ATF4, p-PERK, p-IRE1α in liver tissues of the HFD group was higher than those in the ND group. Moreover, HSP treatment significantly decreased the levels of these molecules in HFD-fed rats, suggesting that HSP can attenuate ERS in the liver.

There are many reasons to suggest that ERS is a critical mediator of proinflammatory response, and extensive findings have confirmed that ERS-induced inflammation contributed to the progression of NAFLD [9, 33]. In this study, it was found that the inflammatory cytokines including IL-1β, IL-6, and TNF-α were all increased in the liver tissues in HFD group compared with those in ND group. In contrast, HSP treatment significantly decreased the expression of the inflammatory cytokines in HFD-fed rats, suggesting that HSP can attenuate ERS-induced inflammation in the liver.

Among the various inflammatory cells implicated in the pathogenesis of NAFLD, macrophages have been shown to play crucial roles in inflammation, which promotes hepatic steatosis in NAFLD [43, 44]. Given that classically activated M1 macrophages showed increased levels of pro-inflammatory cytokines, including interleukin 1-β (IL-1β), tumor necrosis factor α (TNF α), interleukin 6 (IL6), which induce hepatic damage and steatosis [45]. In our study, FFA-stimulated human THP-1 macrophages were employed to establish the *in vitro* NAFLD model [46]. Thapsigargin was used to induce ERS *in vitro* [47]. Western blotting analysis revealed that HSP significantly diminished FFA-induced increase in the protein expression of GRP94, ATF6, p-PERK, and p-IRE1α in human THP-1 cells. Moreover, HSP significantly decreased the protein expression of IL-1β, IL-6, and TNF-α in the FFA-stimulated THP-1 cells. In FFA stimulated THP-1 cells, however, thapsigargin administration significantly increased the expression of inflammatory cytokines, suggesting that HSP could reduce ERS-induced inflammation in FFA stimulated THP-1 macrophages. Immunofluorescence labeling revealed that HSP reduced the levels of GRP94 and p-PERK in FFA-treated THP-1 cells. Furthermore, to confirm the effects of HSP on lipid accumulation, proteins such as SREBP-1c/2 and C/EBP, master transcriptional regulators for lipogenesis that are highly expressed in the liver [47], were tested by western blot, and our results revealed that HSP has significant inhibitory roles for the expression of these proteins, indicating its suppression roles in hepatic lipogenesis, which were reversed by thapsigargin, a powerful ERS inducer (Figure 7).

In conclusion, HSP exhibited an inhibitory effect on ERS-induced inflammation in the liver and THP-1 macrophages, thus restraining NAFLD development. Bioinformatics analysis exposed that inflammatory response and IRE1α activation signaling pathways were enriched in NAFLD. In addition, HSP treatment prevented HFD-induced body weight gain and hepatic steatosis in SD rats; HSP treatment attenuated HFD-induced ERS and inflammation *in vivo* and *in vitro*. These findings implied that HSP may be a promising approach for the treatment of NAFLD.
